# The CORONIS Trial: international study of caesarean section surgical techniques

**DOI:** 10.1186/1745-6215-12-S1-A103

**Published:** 2011-12-13

**Authors:** Edmund Juszczak, Barbara Farrell

**Affiliations:** 1 NPEU Clinical Trials Unit, University of Oxford, Oxford, OX3 7LF, UK

## Objectives

The aim of CORONIS is to examine five specific aspects of caesarean section technique to determine which methods lead to optimum outcomes for women and their babies.

## Background

A variety of surgical techniques for all elements of the caesarean section operation are in use. Many have not yet been rigorously evaluated in RCTs, and it is not known whether any are associated with better outcomes for women and babies.

## Design

CORONIS is a pragmatic multicentre fractional factorial randomised controlled trial and is being conducted in sites in Argentina, Chile, Ghana, India, Kenya, Pakistan and Sudan [1]. Women are eligible if they are undergoing their first or second caesarean section through a transverse abdominal incision. Five comparisons will be carried out using a 2^×5^ balanced incomplete block factorial design. Each woman is allocated THREE of the five pairs of interventions using a bespoke secure web-based randomisation system (with 24/7 automated back-up telephone system) hosted by the NPEU Clinical Trials Unit.

The 5 pairs of interventions are:

i. Blunt versus sharp abdominal entry

ii. Exteriorisation of the uterus for repair versus intra-abdominal repair

iii. Single versus double layer closure of the uterus

iv. Closure versus non-closure of the peritoneum (pelvic and parietal)

v. Chromic catgut versus Polyglactin-910 for uterine repair

Primary outcome: death or maternal infectious morbidity (one or more of the following: antibiotic use for maternal febrile morbidity during postnatal hospital stay, antibiotic use for endometritis, wound infection or peritonitis) or further operative procedures or blood transfusion.

Sample size required: 15,000 women in total; minimum 9,000 women per comparison pair.

## Experience

It is possible to conduct a pragmatic trial in a developing world setting. The six week follow-up rate achieved was 98% despite natural disasters in Pakistan (flooding displacing millions of people) and Chile (earthquake), political unrest in Kenya and Pakistan, and the fact that many women do not have a formal address. Continuous central monitoring of recruitment and trial material usage within sites meant that recruitment targets were met, overall and by pair of interventions. This involved adding new sites in Chile, and switching the allocation used in some sites to compensate for ‘difficulties’ described above. Adherence to the allocation by surgeons has been exceptional. Final analysis of clinical outcomes is underway.

## Conclusions

Good communications (both written and verbal), clear and concise documentation, thorough planning, a strong multi-national collaboration and close working relationships are the building blocks of success.
